# Supplementary material for: Glucose-6-phosphate-dehydrogenase on old peroxisomes maintains self-renewal of epithelial stem cells after asymmetric cell division
Source: Nat Commun. 2025 Apr 26;16:3932. doi: 10.1038/s41467-025-58752-z (PMC12033372; doi:10.1038/s41467-025-58752-z)
Supplement: Supplementary file 1 — Supplementary Information [file 41467_2025_58752_MOESM1_ESM.pdf]

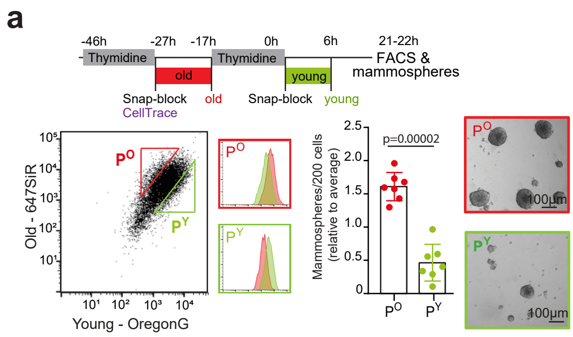

Supplementary fig.1. Old peroxisomes are inherited by self-renewing daughter cells in ACD of human mammary epithelial stem like cells (hMECs)

a, Mammosphere forming capacity of hMECs inheriting old (P^O) or young (P^Y) peroxisomes. Data from seven independent experiments, p-value from paired, two tailed t-test. Data are presented as mean ± SD. Source data are provided as a Source Data file.

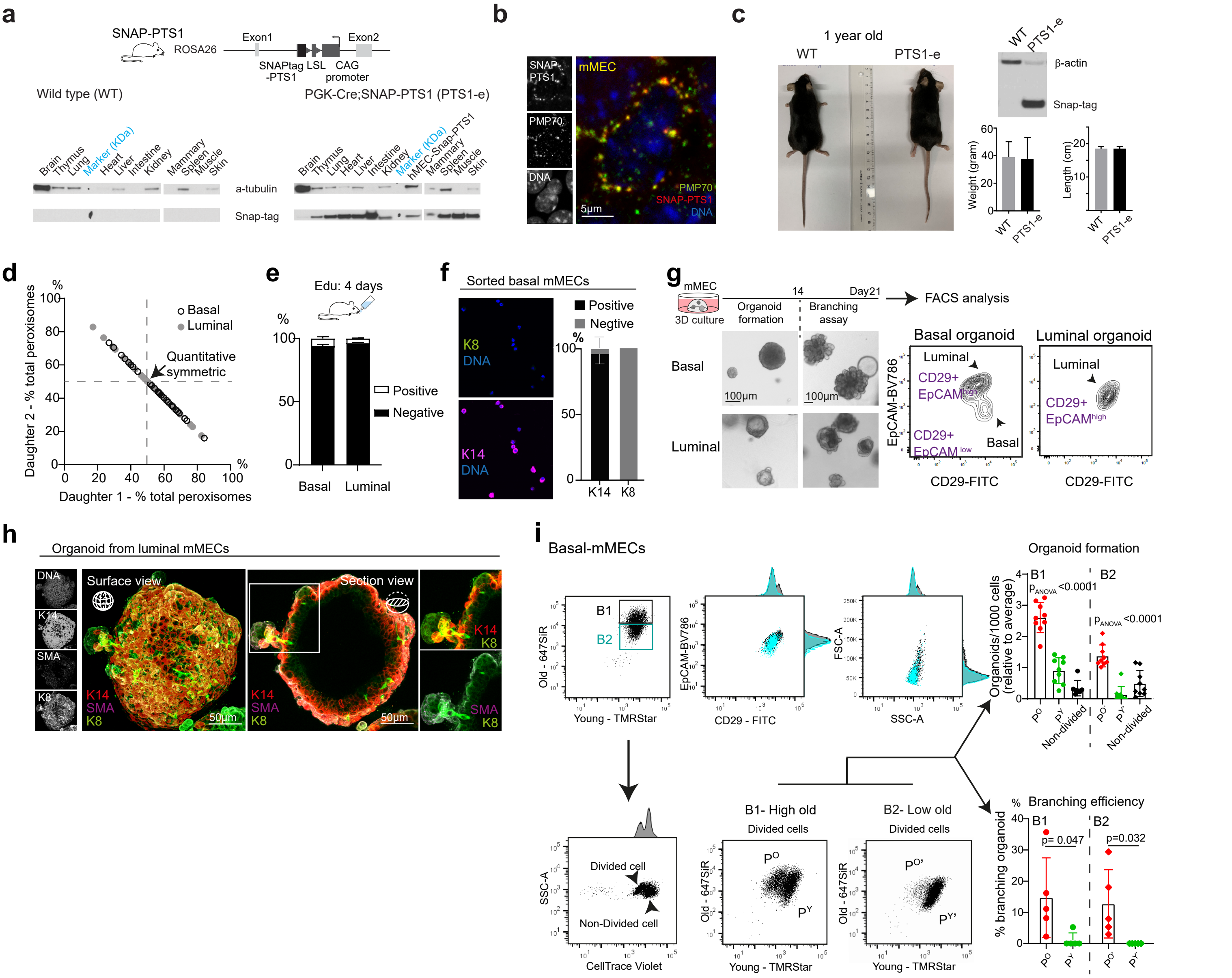

Supplementary fig.2. Old peroxisomes are inherited by the bipotent stem cell in asymmetric division of mouse mammary epithelial basal cells
a, Schematic of knock-in construct used to generate inducible SNAP-PTS1 mouse. Western blot shows expression of SNAPtag-PTS1 and α-tubulin across tissues of PGK-Cre;SNAP-PTS1 (PTS1-e) and wild type (WT) mice. **b**, Representative immunofluorescent staining of PMP70 in mMECs from PTS1-e mice labeled with SNAP-Cell TMR-Star, indicating correct targeting of SNAPtag-PTS1 to peroxisomes *in vivo*. **c**, Normal development and growth of PTS1-e mice. Images, weight, length, and expression of SNAPtag-PTS1 and β-actin in skin of PTS1-e mice compared to WT mice at one year of age are shown. **d**, Quantitative asymmetric apportioning of peroxisomes occurs in both basal (black) and luminal cells (grey). Each datapoint represents a division pair, x- and y-axis showing the percentage of total peroxisomes in each daughter cell during cytokinesis. Data from cells used for Fig.1b. **e**, Percentage of EdU positive and negative basal and luminal mMECs in mice that received EdU in drinking water for four days. 3-5% of both basal and luminal mMECs cycled during the four-day period. Data from 2 mice. **f,** Characterization of FACS sorted basal mMECs. The graph shows percentage of K14 and K8 positive and negative cells in the basal population. No K8 positive cells were observed (0/180). Data from three independent experiments. **g**, Images and analysis of cellular constituents of organoids from basal and luminal mMECs. Only organoids that formed from basal cells were capable of branching. FACS analysis showed that branching organoids originating from basal cells containing both basal and luminal cells, while organoids originating from luminal cells only contained luminal cells. **h**, Surface view and sectional view of an organoid formed by luminal mMECs. Note the lack of branching, disorganized structure, and *in vitro* K14 expression induction of luminal K8-expressing cells. No SMA staining is noted, contrasting organoids formed from basal cells. **i**, Analysis on impact of old peroxisomal quantity prior to asymmetric division. FACS plots show heterogeneity in peroxisome age as well as peroxisomes quantity in the isolated primary basal mMECs. Cells were sub-divided into B1 and B2 containing high or low level of old peroxisomes respectively (data in Fig.1d-g are from B1). Peroxisomal quantity did not impact lineage markers (EpCAM, CD29) and cell size (FSC-A). FACS analysis and sorting strategy is shown for separating basal mMECs based on the inheritance of more old (P^O) or more young (P^Y) peroxisomes after 40h in 2D culture to allow the first division *in vitro* to occur. P^O cells from both B1 or B2 populations had higher organoid formation and branching than P^Y cells. p-value from one-way ANOVA test or two tailed t-test.
Data are presented as mean ± SD. Source data are provided as a Source Data file.

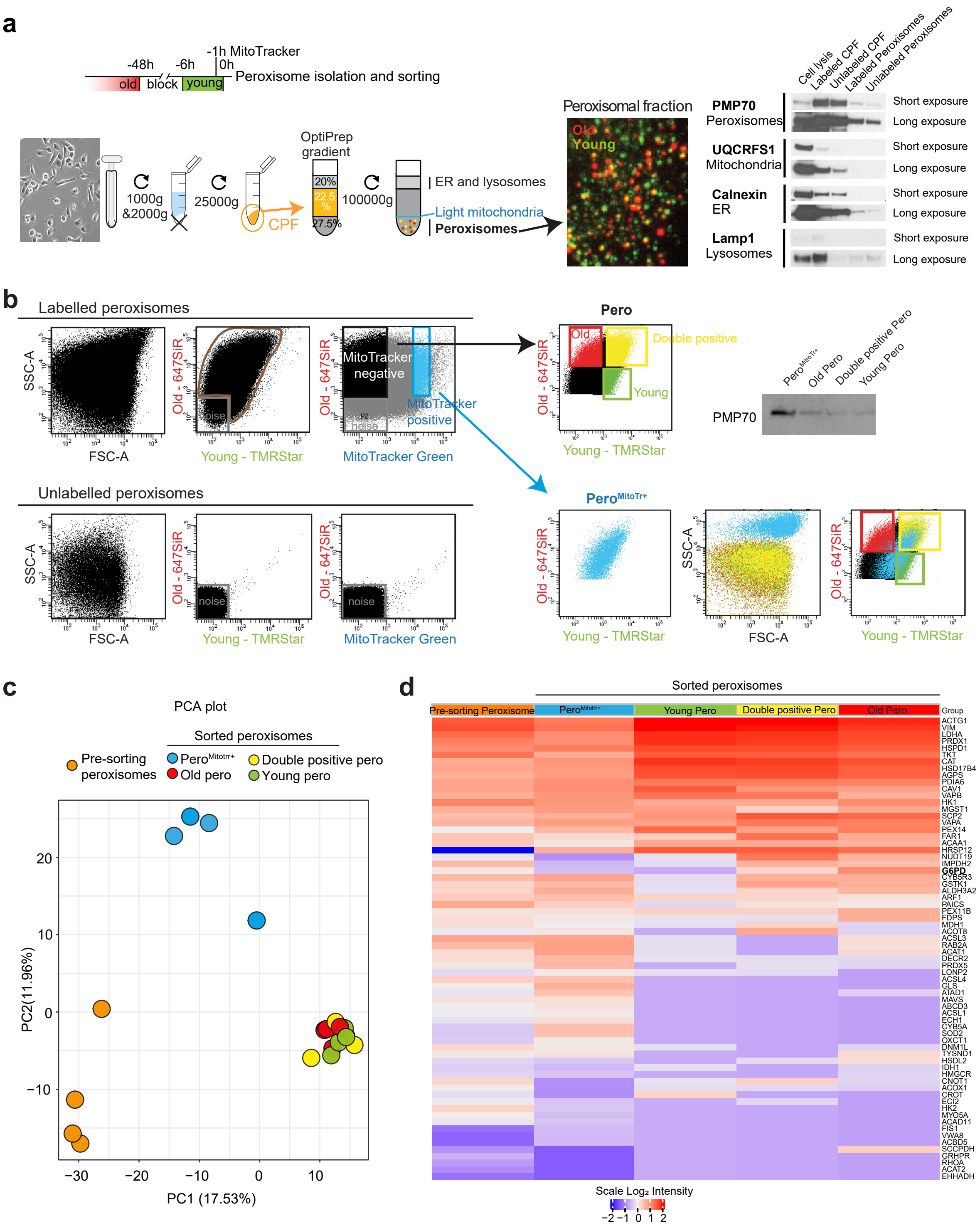

Supplementary fig.3. FACS-mediated single peroxisome sorting for mass spectrometry analysis of different age classes of peroxisomes

a, Schematic of peroxisome labeling and isolation by differential density gradient centrifugation using OptiPrep. Western blot shows level of proteins from other organelles before (Crude Peroxisome Fraction – CPF) and after density gradient centrifugation (Peroxisomes). Unlabeled samples were used as controls for the effect of SNAP-labeling on peroxisome isolation. **b,** FACS-sorting strategy for isolating single peroxisomes for mass spectrometry (MS) analysis. The peroxisome fraction was sub-gated into four different populations for MS: old peroxisomes (old Pero), double positive peroxisomes (double positive Pero), young peroxisomes (young Pero) and Mitotracker positive peroxisomes (Pero$^{Mitotr+}$). Western blot shows the presence of the peroxisomal protein PMP70 in all fractions. The Pero$^{MitoTr+}$ population had a more complex structure (high SSC-A), typical for mitochondria, and was enriched with young SNAPtag-PTS1 and PMP70 proteins. **c,** PCA plot of peroxisomal proteomes of the different fractions in MS analysis. The proteomes of old, young, and double positive peroxisomes were clearly distinct from the Pero$^{MitoTr+}$. **d,** Heatmap showing expression level (average scaled intensity) of detected peroxisomal proteins (source: Uniprot.org) in the different peroxisomal samples, mean of four replicates. See supplementary data 1 for more detail. Source data are provided as a Source Data file.

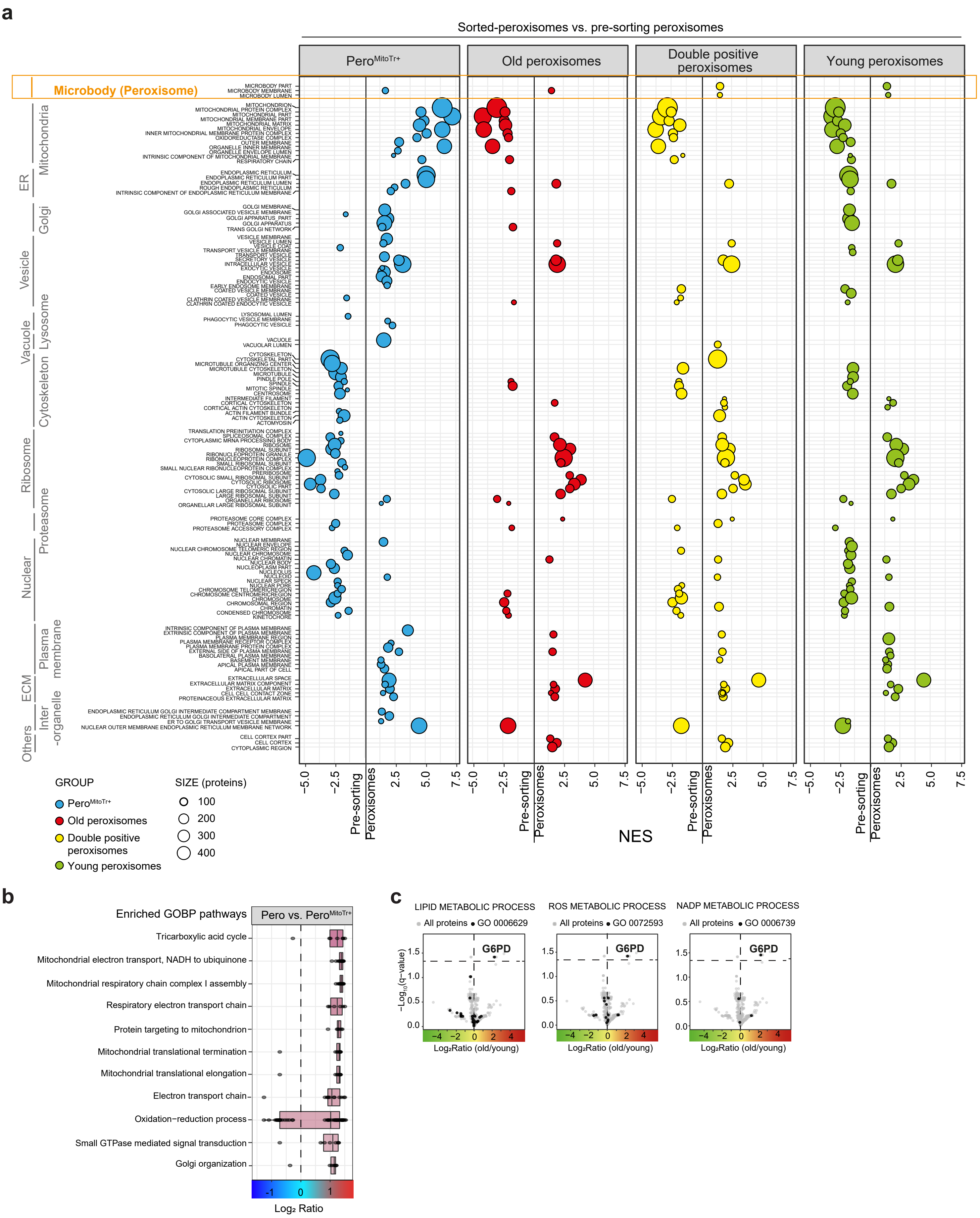

Supplementary fig.4. Proteome of different age classes of peroxisomes

a, GSEA analysis on the enrichments of cellular components in different sorted samples of peroxisomes. Compared to the pre-sorting peroxisomes samples, the rate of peroxisomal proteins in sorted peroxisomes is higher, and $Pero^{MitoTr+}$ population is specifically enriched with proteins associated with ER, Golgi, cellular vesicles and inter-organelle trafficking. GSEA analysis was performed on protein data matrix and Gene Ontology Cellular Component (GOCC; database version 2) and enrichment was tested for each peroxisomal sample against the rest of the dataset. Scoring and metric methods were set to t-test and classic, respectively. Permutation method was set as "gene set". See supplementary data 2 for more detail. **b,** Gene ontology analysis on biological pathways that were enriched in $Pero^{MitoTr+}$ compared to Pero (including old Pero, young Pero and double positive Pero) populations. **c,** Volcano plots of differentially expressed proteins between old and young peroxisomes across the Gene Ontology function annotations related to peroxisomes. Proteins detected in this study are shown in grey, and proteins of a given Gene Ontology class are shown in black. Glucose-6-phosphate-dehydrogenase (G6PD) presented a singular age-selectively enriched exception among proteins involved with peroxisomal metabolic functions.

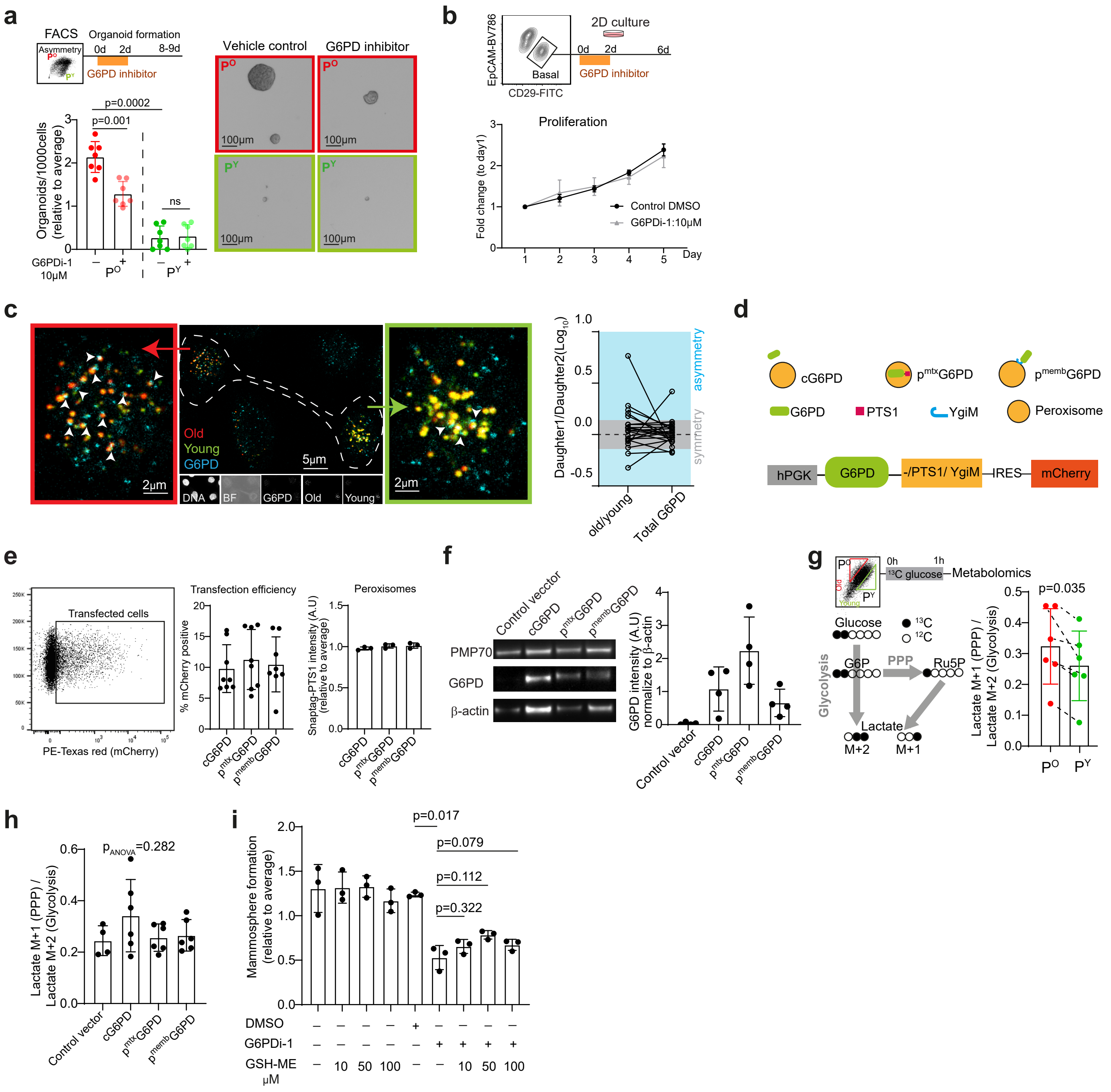

Supplementary fig.5. **Enrichment of G6PD on the peroxisomal membrane influences stemness**

a, Organoid formation by P^O (red) and P^Y (green) daughter cells after transient inhibition of G6PD. G6PD inhibition reduces organoid forming capacity of P^O daughters. Phase contrast for representative organoids treated with G6PD inhibitor (G6PDi) or vehicle control. Data from seven biological replicates. **b**, Proliferation of basal mMECs receiving 10μM G6PDi or DMSO during the first two days of culture. G6PD inhibition did not affect proliferation. **c**, G6PD is primarily located in the cytoplasm, with a portion associated with peroxisomes. During cell division, the total amount of G6PD is not significantly affected by the segregation pattern of peroxisomes. Representative image shows a dividing cell pair, where the cell that inherits more old peroxisomes (highlighted in the red box) does not receive a noticeably higher amount of G6PD (in cyan). The relationship is quantified in the graph on the right: each dot represents a division pair, the line connecting the dots shows the ratio of old to young peroxisomes in relation to the total G6PD ratio between the two daughter cells. **d,** Schematics of overexpression of G6PD in the cytosol (cG6PD), peroxisomal matrix (p^{mtx}G6PD) and on the peroxisomal membrane (p^{memb}G6PD); and the plasmid construct used for overexpression of G6PD. PTS1 is used to target G6PD to the peroxisomal matrix and YgiM is used to target G6PD to the peroxisomal membrane. **e,** FACS strategy for isolating cells that are transfected with G6PD overexpression plasmids. Graphs on the middle and the right show that the transfection efficiency and Snaptag-PTS1 intensity (representing amount of peroxisomes) are similar between cG6PD, p^{mtx}G6PD and p^{memb}G6PD transfected hMECs. **f**, Western blot showing overexpression level of G6PD and quantification of G6PD levels normalized to β-actin. In the control vector, the G6PD sequence is replaced by a luciferase sequence. Data from four independent experiments. **g,** LC-MS analysis of M+1 and M+2 lactate in media secreted from P^O and P^Y hMECs treated with a one-hour pulse of 1,2-^{13}C glucose after FACS isolation. Ratio of M+1 to M+2 isotopomers derived via the pentose phosphate pathway (PPP) or directly from glycolysis respectively shows a modest but significant increase in PPP/glycolysis activity in P^O cells. Data from six independent experiments. **h**, LC-MS analysis of M+1 and M+2 lactate in media secreted from hMECs overexpressing cG6PD, p^{mtx}G6PD, p^{memb}G6PD and control vector treated with a one-hour pulse of 1,2-^{13}C glucose. Ratio of M+1 to M+2 isotopomers derived via the pentose phosphate pathway or directly from glycolysis. Data from six (except four for control vector) independent experiments. **i,** Mammosphere formation of hMECs treated with G6PDi-1 and cell permeable glutathione (GSH-ME). Supplementing hMECs with antioxidant glutathione fails to rescue the impact of inhibiting G6PD activity. Data from three independent replicates.

Data shown as mean ± SD. p-value from paired, two tail t-test or one-way ANOVA test.Source data are provided as a Source Data file.

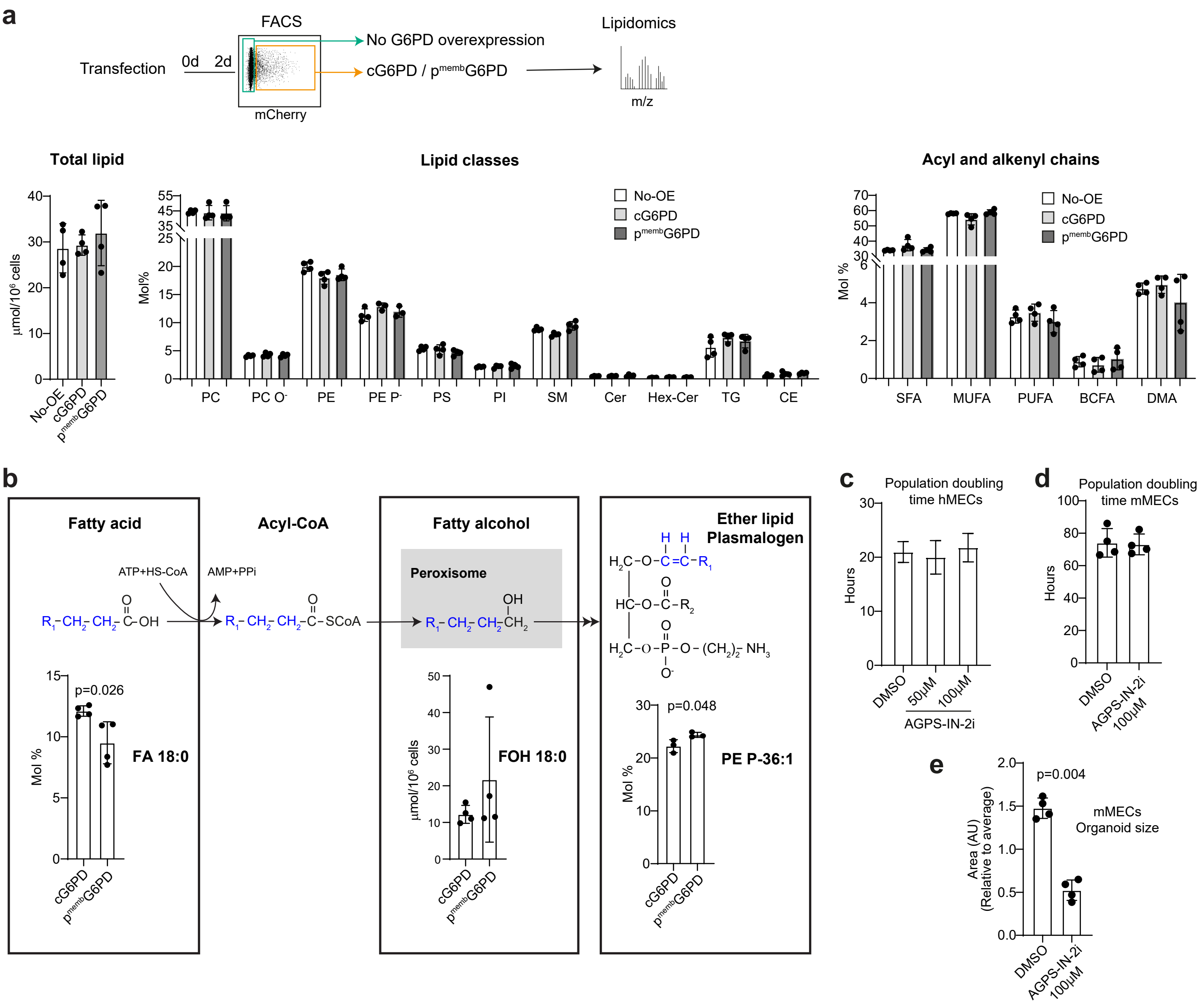

Supplementary fig.6. G6PD on peroxisomal membrane promotes peroxisomal ether lipid synthesis

a, Schematic of FACS isolating cells with and without over expression of cG6PD and p^{memb}G6PD for lipidomic analysis. There were no significant changes in total lipid, major lipid classes and fatty acid between no-overexpression, cG6PD and p^{memb}G6PD hMECs. PC: Phosphatidylcholines, PC O-: Phosphatidylcholine alkyls, PE: Phosphatidylethanolamines, PE P-: Phosphatidylethanolamine plasmalogens, PS: Phosphatidylserines, PI: Phosphatidylinositols, SM: Sphingomyelins, Cer: Ceramides, Hex-Cer: Hexosylceramides, TG: Triacylglycerols, CE: Cholesteryl esters, SFA: Saturated fatty acids, MUFA: Monounsaturated fatty acids, PUFA: Polyunsaturated fatty acids, BCFA: Branched-chain fatty acids, DMA: Dimethyl acetals. Data from four (except three for PE P-) independent replicates, p-value from two tail t-test. **b,** Illustration of the process of ether lipid synthesis and lipidomic analysis focusing on stearic acid (FA 18:0) involved in ether lipid synthesis. Compared to cG6PD, the lipidome of p^{memb}G6PD showed a significant decrease in stearic acid (FA 18:0), and a significant increase of plasmalogen PE P- 36:1 (major PE P- species being predominantly PE P-18:0/18:1). Data from four (except three for PE P-) independent replicates, p-value from two tail t-test. **c,d,** The population doubling time of hMECs (c) and mMECs (d) are not affected by the dosages of AGPS inhibitor, AGPS-IN-2i, used. **e,** AGPS-IN-2i significantly reduces the size of organoids formed by mMECs. Data from four biological replicates, p-value from paired, two tail t-test.

Data shown as mean ± SD. Source data are provided as a Source Data file.

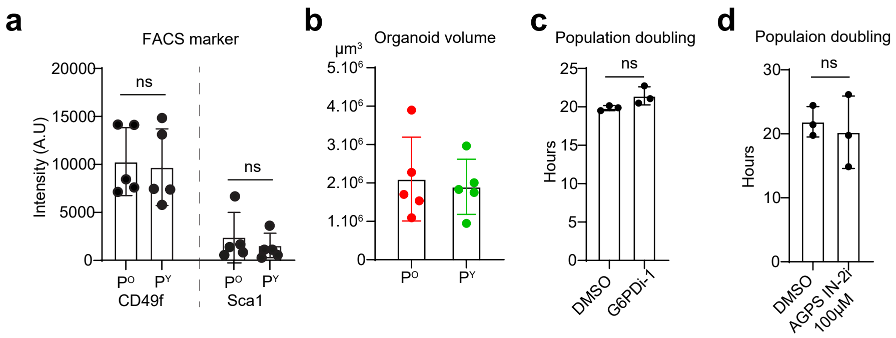

Supplementary fig.7. Age-selective apportioning of peroxisomes in EpSCs *in vivo*

a, Intensity of CD49f and Sca1, markers for EpSCs, in P^O and P^Y cells before organoid formation assays in fig. 3c. P^O and P^Y have similar level of CD49f and Sca1. **b,** Volume of organoids from P^O and P^Y of EpSCs in Fig.3d. Data from five biological replicates, p-value from paired, two tailed t-test. **c**, Population doubling time of EpSCs receiving 10µM G6PDi-1 or DMSO during the first two days of culture. G6PD inhibition did not affect proliferation of EpSCs. **d,** Population doubling time of EpSCs treated with 100µM AGPS-IN-2i or DMSO during the first two days of culture. AGPS inhibition did not affect proliferation of EpSCs. Data from three independent experiments, p-value from paired, two tailed t-test.
Data shown as mean ± SD. Source data are provided as a Source Data file.
