# Supplementary material for: Glucose-6-phosphate-dehydrogenase on old peroxisomes maintains self-renewal of epithelial stem cells after asymmetric cell division
Source: Nat Commun. 2025 Apr 26;16:3932. doi: 10.1038/s41467-025-58752-z (PMC12033372; doi:10.1038/s41467-025-58752-z)
Supplement: Supplementary file 2 — Description of Additional Supplementary Information [file 41467_2025_58752_MOESM2_ESM.docx]

**Description of Additional Supplementary Files**

File Name: Supplementary Data 1

Description: The proteomes of old, young and double positive peroxisomes.

File Name: Supplementary Data 2

Description: Gene set enrichment analysis on the enriched cellular components in different sorted peroxisome samples.
