# Supplementary material for: Glucose-6-phosphate-dehydrogenase on old peroxisomes maintains self-renewal of epithelial stem cells after asymmetric cell division
Source: Nat Commun. 2025 Apr 26;16:3932. doi: 10.1038/s41467-025-58752-z (PMC12033372; doi:10.1038/s41467-025-58752-z)
Supplement: Supplementary file 5 — Reporting Summary [file 41467_2025_58752_MOESM5_ESM.pdf]

nature portfolio

Corresponding author(s): Pekka Katajisto

Last updated by author(s): Feb 25, 2025

Reporting Summary

Nature Portfolio wishes to improve the reproducibility of the work that we publish. This form provides structure for consistency and transparency in reporting. For further information on Nature Portfolio policies, see our Editorial Policies and the Editorial Policy Checklist.

Statistics

For all statistical analyses, confirm that the following items are present in the figure legend, table legend, main text, or Methods section.

n/a	Confirmed	
☐	☒	The exact sample size (*n*) for each experimental group/condition, given as a discrete number and unit of measurement
☒	☐	A statement on whether measurements were taken from distinct samples or whether the same sample was measured repeatedly
☐	☒	The statistical test(s) used AND whether they are one- or two-sided<br>*Only common tests should be described solely by name; describe more complex techniques in the Methods section.*
☒	☐	A description of all covariates tested
☒	☐	A description of any assumptions or corrections, such as tests of normality and adjustment for multiple comparisons
☐	☒	A full description of the statistical parameters including central tendency (e.g. means) or other basic estimates (e.g. regression coefficient) AND variation (e.g. standard deviation) or associated estimates of uncertainty (e.g. confidence intervals)
☐	☒	For null hypothesis testing, the test statistic (e.g. *F*, *t*, *r*) with confidence intervals, effect sizes, degrees of freedom and *P* value noted<br>*Give P values as exact values whenever suitable.*
☒	☐	For Bayesian analysis, information on the choice of priors and Markov chain Monte Carlo settings
☒	☐	For hierarchical and complex designs, identification of the appropriate level for tests and full reporting of outcomes
☒	☐	Estimates of effect sizes (e.g. Cohen's *d*, Pearson's *r*), indicating how they were calculated

Our web collection on statistics for biologists contains articles on many of the points above.

Software and code

Policy information about availability of computer code

Data collection	BD FACS Diva; LAS X; Zen Black v2.1; Slide Book 5.5, Bio-Rad CFX
Data analysis	Fiji ImageJ 1.53; Imaris x64; FlowJo V10; Graphpad Prism 8; Andromeda search engine/Maxquant software suite (v1.6.14.0); Microsoft Excel (Office 16)

For manuscripts utilizing custom algorithms or software that are central to the research but not yet described in published literature, software must be made available to editors and reviewers. We strongly encourage code deposition in a community repository (e.g. GitHub). See the Nature Portfolio guidelines for submitting code & software for further information.

Data

Policy information about availability of data

All manuscripts must include a data availability statement. This statement should provide the following information, where applicable:

- Accession codes, unique identifiers, or web links for publicly available datasets
- A description of any restrictions on data availability
- For clinical datasets or third party data, please ensure that the statement adheres to our policy

The mass spectrometry proteomics data has been deposited to the ProteomeXchange Consortium via the PRIDE partner repository with the dataset identifier PDX028679.

Research involving human participants, their data, or biological material

Policy information about studies with human participants or human data. See also policy information about sex, gender (identity/presentation), and sexual orientation and race, ethnicity and racism.

Reporting on sex and gender	*Use the terms sex (biological attribute) and gender (shaped by social and cultural circumstances) carefully in order to avoid confusing both terms. Indicate if findings apply to only one sex or gender; describe whether sex and gender were considered in study design; whether sex and/or gender was determined based on self-reporting or assigned and methods used. Provide in the source data disaggregated sex and gender data, where this information has been collected, and if consent has been obtained for sharing of individual-level data; provide overall numbers in this Reporting Summary. Please state if this information has not been collected. Report sex- and gender-based analyses where performed, justify reasons for lack of sex- and gender-based analysis.*
Reporting on race, ethnicity, or other socially relevant groupings	*Please specify the socially constructed or socially relevant categorization variable(s) used in your manuscript and explain why they were used. Please note that such variables should not be used as proxies for other socially constructed/relevant variables (for example, race or ethnicity should not be used as a proxy for socioeconomic status). Provide clear definitions of the relevant terms used, how they were provided (by the participants/respondents, the researchers, or third parties), and the method(s) used to classify people into the different categories (e.g. self-report, census or administrative data, social media data, etc.) Please provide details about how you controlled for confounding variables in your analyses.*
Population characteristics	*Describe the covariate-relevant population characteristics of the human research participants (e.g. age, genotypic information, past and current diagnosis and treatment categories). If you filled out the behavioural & social sciences study design questions and have nothing to add here, write "See above."*
Recruitment	*Describe how participants were recruited. Outline any potential self-selection bias or other biases that may be present and how these are likely to impact results.*
Ethics oversight	*Identify the organization(s) that approved the study protocol.*

Note that full information on the approval of the study protocol must also be provided in the manuscript.

Field-specific reporting

Please select the one below that is the best fit for your research. If you are not sure, read the appropriate sections before making your selection.

☒ Life sciences ☐ Behavioural & social sciences ☐ Ecological, evolutionary & environmental sciences

For a reference copy of the document with all sections, see nature.com/documents/nr-reporting-summary-flat.pdf

Life sciences study design

All studies must disclose on these points even when the disclosure is negative.

Sample size	Sample size was determined based on the number of independent experiment or biological sample (i.g. each mouse is a biological sample). No statistical method was used to predetermine the sample size. The sample size was determined based on the minimum number required for the statistical test (minimum 3 replicates).
Data exclusions	No data exclusion
Replication	Replication was counted base one independent experiments:<br>- Experiment with cell line: experiment happened in different day.<br>- Experiment with animal: replicate was count as different mouse. Data are at least from 2 different days of experiments
Randomization	For experiments that had comparison between treated and control (i.g. G6PD inhibitor, AGPS inhibitor treatment), the starting materials was divided equally into wells/dishes and randomly chosen for control or treatment.
Blinding	Researcher was not blinding during data collection and analysis

Reporting for specific materials, systems and methods

We require information from authors about some types of materials, experimental systems and methods used in many studies. Here, indicate whether each material, system or method listed is relevant to your study. If you are not sure if a list item applies to your research, read the appropriate section before selecting a response.

Materials & experimental systems

n/a	Involved in the study	
☐	☒	Antibodies
☐	☒	Eukaryotic cell lines
☒	☐	Palaeontology and archaeology
☐	☒	Animals and other organisms
☒	☐	Clinical data
☒	☐	Dual use research of concern
☒	☐	Plants

Methods

n/a	Involved in the study	
☒	☐	ChIP-seq
☐	☒	Flow cytometry
☒	☐	MRI-based neuroimaging

Antibodies

Antibodies used	CD29 FITC (Miltenyi Biotec, 130-102-975), CD326 (Ep-CAM) BV786 (BD; 740958), CD45 PerCP-Cy5.5 (Tonbo Biosciences; 65-0452-U100), CD31 PerCP-Cy5.5 (BD; 562861), Ter-119 PerCP-Cy5.5 (BD; 560512), mouse monoclonal anti-PMP70 antibody (Sigma-Aldrich, SAB4200181); rabbit anti-catalase antibody (D4P7B) (Cell Signaling Technology, 12980); mouse a-tubulin (DM1A) antibody (Cell Signaling Technology, 3873); anti-SNAP-tag antibody (New England BioLabs, P9310S); anti-beta Actin antibody (Abcam, ab8227); Anti-UQCRFS1 [EPR16288] (Abcam, ab191078, rabbit); anti-LAMP1 antibody (Abcam, ab24170); Calnexin polyclonal antibody (Enzo, ADI-SPA-860), anti-mouse IgG HRP-linked antibody (Cell Signaling Technology, 7076S) or anti-rabbit IgG HRP-linked antibody (Sigma-Aldrich, A0545), rabbit polyclonal anti keratin 14 antibody (BioLegend, 905301); monoclonal anti-Actin, a-smooth muscle (Sigma-Aldrich, A2547), anti-cytokeratin 8 antibody (TROMA-I, DSHB); anti-Glucose 6 Phosphate Dehydrogenase antibody (Abcam, ab133525); anti-E-cadherin (BD, 610182), anti-alpha tubulin (Abcam, ab18251), goat anti rabbit IgG (H+L) Alexa Flour 488 antibody (Invitrogen, A11008), chicken anti-mouse Alexa Flour 488 antibody (Life Technologies, A21200), chicken anti-rat IgG Alexa Flour 488 antibody (Life Technology, A21470), goat anti-mouse Alexa Fluor 488 antibody (Invitrogen, A-11029), Goat anti-rabbit IgG Alexa Fluor 594 antibody (Life Technologies, A11012), Alexa flour donkey anti-mouse 594 antibody (Life Technologies, A21203), Goat anti-rabbit IgG Alexa flour 647 antibody (Life Technologies, A21244); Alexa flour goat anti-mouse 647 IgG (H+L) antibody (Life Technologies, A21235), goat anti-Rat IgG Alexa Flour 633 (Invitrogen, A-21094).
Validation	Validation from manufacturers or lab with negative/positive control

Eukaryotic cell lines

Policy information about cell lines and Sex and Gender in Research

Cell line source(s)	hMEC line FL2 (Chaffer et al., 2011); hMEC SNAP-PTS1 (this paper);
Authentication	No authentication
Mycoplasma contamination	No Mycoplasma contamination (tests were done in the laboratory)
Commonly misidentified lines (See ICLAC register)	No commonly misidentified lines

Animals and other research organisms

Policy information about studies involving animals; ARRIVE guidelines recommended for reporting animal research, and Sex and Gender in Research

Laboratory animals	SNAP-PTS1 mouse (MGI:6466977) with Black6 background. Mammary glands were collected from female (11-16 weeks old, virgin females). Skin samples were collected from pups (day 3)
Wild animals	No wild animal
Reporting on sex	For primary mammary epithelial stem cells: mice were females.<br>For skin tissues and cells: there was no sex based analysis. Pups under 3 days old were used. Sex of animal should not effect the skin cells behavior during this time of development.
Field-collected samples	No field collected samples
Ethics oversight	Animal housing and experiments were done in accordance to Finnish National Animal Experimentation Board with ethical approval

Note that full information on the approval of the study protocol must also be provided in the manuscript.

Plants

Seed stocks

Report on the source of all seed stocks or other plant material used. If applicable, state the seed stock centre and catalogue number. If plant specimens were collected from the field, describe the collection location, date and sampling procedures.

Novel plant genotypes

Describe the methods by which all novel plant genotypes were produced. This includes those generated by transgenic approaches, gene editing, chemical/radiation-based mutagenesis and hybridization. For transgenic lines, describe the transformation method, the number of independent lines analyzed and the generation upon which experiments were performed. For gene-edited lines, describe the editor used, the endogenous sequence targeted for editing, the targeting guide RNA sequence (if applicable) and how the editor was applied.

Authentication

Describe any authentication procedures for each seed stock used or novel genotype generated. Describe any experiments used to assess the effect of a mutation and, where applicable, how potential secondary effects (e.g. second site T-DNA insertions, mosiacism, off-target gene editing) were examined.

Flow Cytometry

Plots

Confirm that:

- [x] The axis labels state the marker and fluorochrome used (e.g. CD4-FITC).
- [x] The axis scales are clearly visible. Include numbers along axes only for bottom left plot of group (a 'group' is an analysis of identical markers).
- [x] All plots are contour plots with outliers or pseudocolor plots.
- [ ] A numerical value for number of cells or percentage (with statistics) is provided.

Methodology

Sample preparation

1. Isolation mouse mammary epithelial cells

mMECs was isolated from 11-16 weeks old virgin females. Mammary glands (pairs 2-3 and 4-5) were dissected, finely cut and incubated in 0.01mg of Collagenase A per 1g of tissue in mMEC growth media (Advance DMEM/F12 (Life Technologies, 12634028) containing 10% FBS (Gibco, 10270106), 5 ng/ml mEGF (R&D Systems, 2028-WG-200), 5 µg/ml insulin (Sigma-Aldrich, I9278), 1 µg/ml hydrocortisone (Sigma-Aldrich, H4001), 2 mM glutamine (Sigma-Aldrich, 90114C), 50 µg/ml penicillin and streptomycin) with 10mM Hepes (Sigma-Aldrich, H3375) shaking at 120rpm for 2hours at 37oC. The cell suspension was centrifuged for 10min at 400xg to collect the pellet followed by 2-3 pulse centrifugations at 400xg to enrich for mMECs. Next, cell pellets were treated with 0.05% Trypsin-EDTA (DIFCO, J.T.BAKER) for 7-10 minutes, the cell suspension was filter through a 70µm cell strainer and centrifuged at 300xg for 5 minutes. The cell pellet was stained with the following antibodies: CD29 FITC (Miltenyi Biotec, 130-102-975), CD326 (Ep-CAM) BV786 (BD; 740958), CD45 PerCP-Cy5.5 (Tonbo Biosciences; 65-0452-U100), CD31 PerCP-Cy5.5 (BD; 562861), Ter-119 PerCP-Cy5.5 (BD; 560512) for 30 minutes on ice (1:500 dilution for all antibodies). After washing with Advance DMEM/F12 base media, cells were resuspended in mMEC growth media with 7AAD (Life Technologies, A1310, 1µg/ml) for at least 5 minutes on ice before sorting.

2. Basal mMECs organoid (3D) culture

After in vivo labeling, isolation and sorting, basal mMECs were labeled with CellTrace Violet (1:1,000 dilution) in MEGM medium (Lonza, CC-3153) according to the manufacturer's instructions. After washing, cells were cultured in mMEC single cell culture media (mMEC growth media supplement with 500ng/ml R-spondin (R&D Systems, 3474-RS), 100ng/ml Noggin (PeproTech, 250-38-250ug) on fibronectin coated 12-well plates for 40 hours at 37oC, 5% CO2 to allow cell division in vitro. Cells were treated with trypsin to detach and collected in mMEC single cell culture media with 7AAD (Life Technologies, A1310, 1µg/ml) for at least 5 minutes before sorting for 3D cultur

3. Isolation human mammary epithelial cells

hMECs were synchronized with a double thymidine block 10,11. Labeling was done at 37oC, 5% CO2 as described above. In short, cells were treated with 5mM thymidine (Sigma-Aldrich, T1895) for 19 hours, washed with PBS, detached with 0.05% trypsin-EDTA and collected by centrifugation (400xg for 5 minutes). Cells were treated with SNAP-Cell Block (4.5µM) for 30 minutes. SNAP-Cell block was then diluted with base DMEM media (Sigma-Aldrich, D7777, at least 10X labeling volume) cells were collected by centrifugation and stained with CellTrace Violet (Thermo Fisher Scientific, C34571) according to manufacturer's instructions. After centrifugation, cells were plated in MEGM. Labeling of old peroxisomes with SNAP-Cell Sir647 (1.5µM, 30 minutes, 37oC, 5%CO2) was performed on dishes 9 hours after releasing from first thymidine followed by a 30 minutes wash with MEGM and second thymidine treatment. Cells were released from second thymidine after 17 hours followed by treatment with SNAP-Cell Block (3µM, 30 minutes) and 30 minutes washes in MEGM. Young peroxisomes were labeled with SNAP-Cell Oregon Green (1.5µM, 30 minutes) 6 hours after second thymidine release. At 21-22 hours after second thymidine release, cells were detached with 0.05% trypsin-EDTA and collected for cell sorting by centrifugation at 400xg for 5 minutes.

4. Single organelle sorting

SNAP labeling peroxisomes was done in intact hMECs as described above with timeline indicated in the figure. Briefly hMECs were cultured in MEGM media for 48 hours and detached with trypsin-EDTA. After centrifugation, cells were treated with 1.5µM SNAP-Cell Sir647 for old peroxisomes followed by 30 minutes wash in MEGM. Cells were plated in MEGM for 42 hours followed by SNAP-Cell block (3µM). Cells were then detached with trypsin-EDTA and collected by centrifugation for young peroxisome labeling with SNAP-Cell TMRstar (1.5µM). After young peroxisome labeling, cells were treated with 150nM MitoTracker Green FM (Thermo Fisher Scientific, M7514) for 30 minutes. Cells were collected by centrifugation for peroxisome isolation.

Peroxisomes were isolated using Peroxisome Isolation Kit (Sigma-Aldrich, PEROX1) according to the manufacture's

	instruction. Briefly, cells were homogenized in 1X peroxisome extraction buffer (provided with the kit) by 7ml Dounce glass tissue grinder (Sigma-Aldrich, T0566) with small clearance pestle (Sigma-Aldrich, P1235), 20-25 strokes. Samples were centrifuged at 1,000xg and then 2,000xg for 10 minutes, and the supernatant was centrifuged at 25,000xg for 20 minutes. The pellet was collected and suspended in peroxisome extraction buffer using pellet pestle (Sigma-Aldrich, Z35,994-7; Z35,997-1) to obtain a crude peroxisome fraction (CPF). The CPF was mixed with Optiprep Density gradient medium and Optiprep dilution buffer (provided with the kit) to make an Optiprep concentration of 22.5%. The 22.5% mixture was place in between 27.5% and 20% Optiprep solution in a centrifugation tube (Berkman Coulter, 343778) and centrifuged for 1.5 hours at 100,000xg (Optima MAX Ultracentrifuge, TLA 120.2 rotor, Berkman Coulter). The top layers of 20% and 22.5% and the interface at the 22.5%/27.5% was aspirated off. The bottom layer containing the peroxisome fraction was collected and placed on ice for single organelle sorting.
Instrument	BD FACSAria II Cell sorter (Lasers: Near UV 375nm, Blue 488nm, Red 633nm) or BD FACSAria Fusion Flow Cytometer (Laser 405nm, 488nm, 561nm, 633nm)
Software	BD FASC Diva of FlowJo (for ananlysis)
Cell population abundance	For mMECs: Around 20-30% of the FACS samples are live Luminal and basal cells mMECs. Luminal/basal ratio range from 0.4-2,5/3.<br>- For daughter cells that inherited old or young peroxisomes: each population is around 20% of the mother population
Gating strategy	1. mMECs :<br>- The basal mMECs were identified with 7AAD-, Lin (CD31, CD45, Ter-119)-, EpCAMmed, CD29high and luminal mMECs were identified with 7AAD-, Lin (CD31, CD45, Ter-119)-, EpCAMhigh, CD29low.<br>- Cells that had divided (7AAD-, CellTrace Violetlow) were sub-gated into PO and PY populations based on the amount of old and young peroxisomes.<br>2.hMECs<br>Cells that had divided were recognized by low CellTrace Violet staining (DAPI channel) and were sub-gated into PO and PY populations based on the amount of old and young peroxisomes (APC and FITC channel).<br>3. Single peroxisome FACS<br>SNAP-Cell Sir-647, SNAP-Cell TMR Star and MitoTracker Green were detected with APC, PE and FITC channels respectively. APC and PE channels (for detecting old and young peroxisomes) were used for setting the thresholds at 500. Gating into populations was based on the amount of old and young peroxisomes and mitotracker green<br>4. EpSCs<br>The EpSCs were identified as Sytox blue-, CD49high and Sca1high. EpSCs were sub-gated into PO and PY populations based on the amount of old and young peroxisomes

☒ Tick this box to confirm that a figure exemplifying the gating strategy is provided in the Supplementary Information.
